# Reduction of the Powerful Greenhouse Gas N_2_O in the South-Eastern Indian Ocean

**DOI:** 10.1371/journal.pone.0145996

**Published:** 2016-01-22

**Authors:** Eric J. Raes, Levente Bodrossy, Jodie Van de Kamp, Bronwyn Holmes, Nick Hardman-Mountford, Peter A. Thompson, Allison S. McInnes, Anya M. Waite

**Affiliations:** 1 The Oceans Institute, University of Western Australia, M047 35 Stirling Hwy Crawley, 6009 WA, Australia; 2 CSIRO Oceans and Atmosphere Flagship, GPO Box 1538, Hobart, 7001 TAS, Australia; 3 CSIRO Oceans and Atmosphere Flagship, Private Bag 5, Wembley, 6913 WA, Australia; 4 University of Technology, Sydney, Plant Functional Biology & Climate Change, City campus 15 Broadway Ultimo NSW 2007, Australia; 5 Alfred Wegener Institute for Polar and Marine Research, Am Handelshafen 12, 27570 Bremerhaven, Germany; CAS, CHINA

## Abstract

Nitrous oxide (N_2_O) is a powerful greenhouse gas and a key catalyst of stratospheric ozone depletion. Yet, little data exist about the sink and source terms of the production and reduction of N_2_O outside the well-known oxygen minimum zones (OMZ). Here we show the presence of functional marker genes for the reduction of N_2_O in the last step of the denitrification process (nitrous oxide reductase genes; *nosZ*) in oxygenated surface waters (180–250 O_2_ μmol.kg^-1^) in the south-eastern Indian Ocean. Overall copy numbers indicated that *nosZ* genes represented a significant proportion of the microbial community, which is unexpected in these oxygenated waters. Our data show strong temperature sensitivity for *nosZ* genes and reaction rates along a vast latitudinal gradient (32°S-12°S). These data suggest a large N_2_O sink in the warmer Tropical waters of the south-eastern Indian Ocean. Clone sequencing from PCR products revealed that most denitrification genes belonged to *Rhodobacteraceae*. Our work highlights the need to investigate the feedback and tight linkages between nitrification and denitrification (both sources of N_2_O, but the latter also a source of bioavailable N losses) in the understudied yet strategic Indian Ocean and other oligotrophic systems.

## Introduction

Emissions of nitrous oxide (N_2_O) are of an eminent concern as the greenhouse warming power is 300 times stronger than CO_2_ [1,2]. N_2_O is the precursor of nitric oxide (NO) radicals and the single most destructive source of ozone-depleting [3]. Marine N_2_O production is predicted to increase under global warming scenarios including ocean acidification, sea surface warming and coastal eutrophication [1]. Yet limited data exists on potential feedback systems in the marine environment.

**Fig 1:**
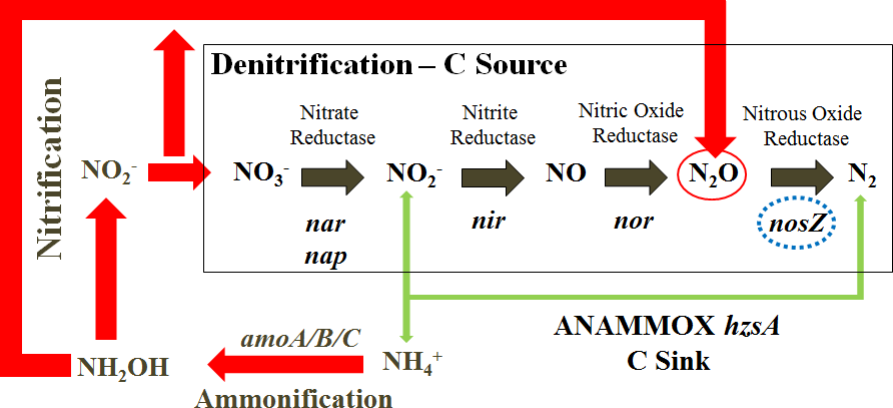
N–cycle emphasising the production of N_2_O through denitrification (grey arrows) and nitrification (red arrows). The production of N_2_ through anammox is presented with green arrows. Arrow width indicates suggested pathway importance in the south-eastern Indian Ocean. Marker genes for the different steps of the nitrogen cycle are in italics. The nitrous oxide reductase gene (*nosZ*) is highlighted with a dashed blue circle. Heterotrophic denitrification (*nir*/nor genes) is highlighted as a C source while the autotrophic anammox process (*hzsA* genes) is highlighted as a C sink. Note: dissimilatory reduction of NO_2_^−^ to NH_4_^+^ and N_2_O is not presented on this figure. Figure adapted from Throbäck et al. [53]

N_2_O production occurs during nitrification both during the formation of hydroxylamine from NH_4_^+^ and during the formation of NO_3_^-^ from NO_2_^-^ (Fig 1). The production of N_2_O is oxygen sensitive and nitrification rates are predicted to increase by the expansion of low oxygen waters [4]. Hypoxic and suboxic waters (<50 μmol.L^-1^ and <5 μmol.L^-1^ Voss et al. [5],[6]) have been expanding over a 50 year period in regions from the subarctic [7] to the tropical oceans [8] and are hot spots for nitrification and denitrification (fixed N removal) processes. In oxygen minimum zones (OMZ) such as those in the Arabian Sea [9,10], off the coast of Peru [11] and in the Benguela upwelling waters [4] the respiratory activity of heterotrophic denitrifying bacteria have been shown to contribute up to 35% of the N_2_O budget [12].

Waite et al. [13] noted that the surface waters at low-latitude in the South Indian Ocean are depleted in oxygen (“NO” values as low as 175 μmol kg^-1^) compared to the open Atlantic and Pacific [14]. These lower oxygenated waters and their predicted expansion are a hot spot for nitrification [15] and consequently for N_2_O production. Nitrogen inputs, including the redistribution of fixed N_2_ through ammonification [16] and nitrification [17] have been shown to alter over relatively short timescales and fuel primary productivity on a regional scale in the south-eastern Indian Ocean [18,19]. The pivotal role of nitrification in this ocean basin leaves a big question mark on the magnitude of N_2_O production under current and future climate scenarios.

The only known metabolic pathway that converts the destructive N_2_O gas into the inert N_2_ gas is through the copper-containing enzyme nitrous oxide reductase (*nosZ*). The *nosZ* enzyme is found in most denitrifying organisms and also in a few non-denitrifying bacteria, such as Vibrio succinogenes [20]. If non-denitrifying bacteria are dominant then the south-eastern Indian Ocean can act as sink for N_2_O gas. Denitrification and the consequent bio-available N-losses can still occur when the former dominate the ecosystem. The significance of accurately quantifying *nosZ* genes are crucial to give us a better insights in the sinks and sources terms of N_2_O production and reduction.

## Material and Methods

### PCR, real-time PCR and clone library analysis

**Fig 2:**
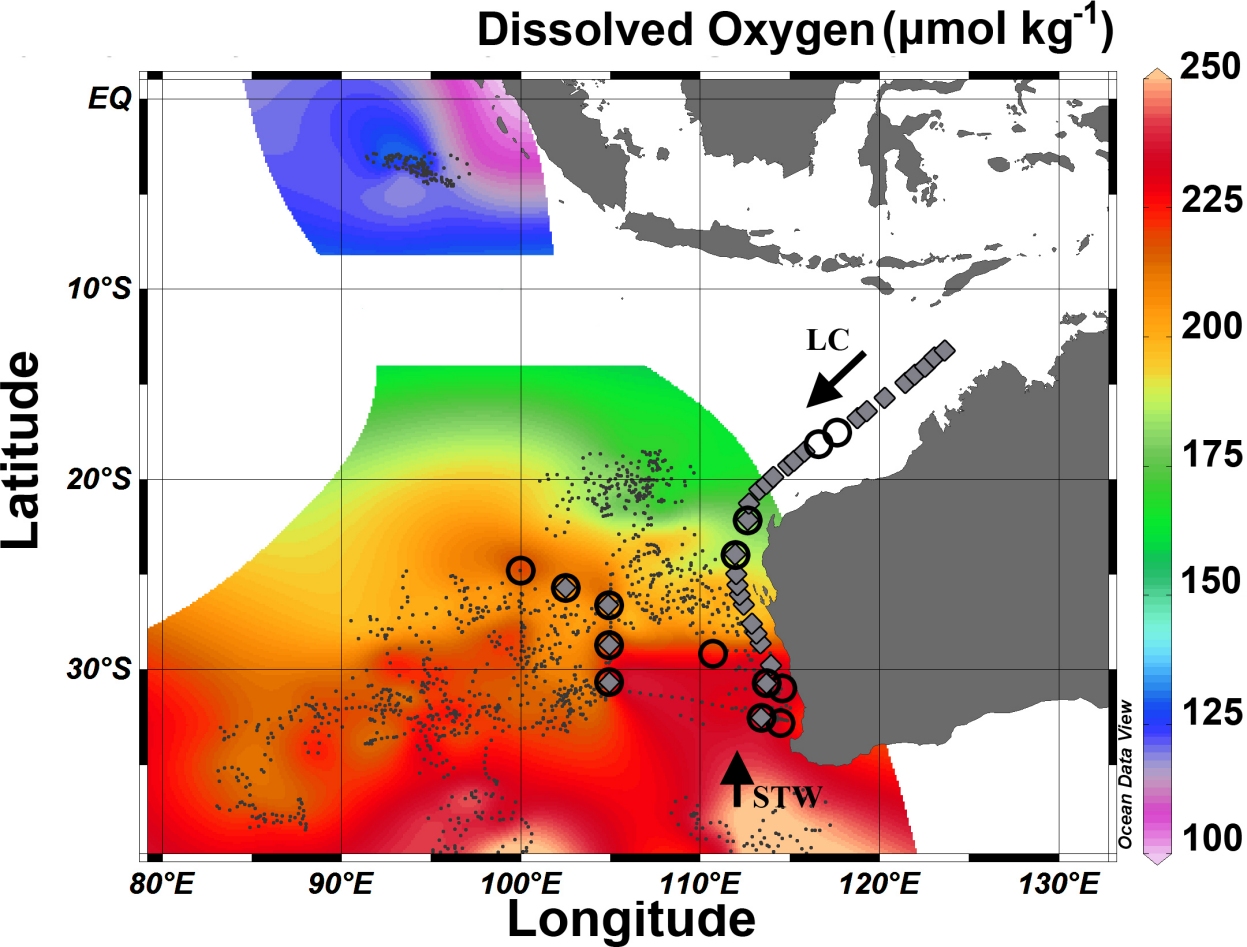
Spatial extent of oxygen concentrations (integrated from the surface to 200m depth). Data (black squares) sourced from all available Argo floats with oxygen sensor in the region and cruise voyages conducted on the RV Southern Surveyor in 2010, 2011, 2012 and 2013. Low dissolved oxygen (<125 μmol.kg^-1^) stretches from the tropics to the subtropics. Black circles highlight sampling stations for functional N genes (*nosZ* and *hzsA*). Grey diamonds denote sampling station for heterotrophic microbial counts. Regional water masses Subtropical waters (STW) and Leeuwin Current waters (LC) are denoted by black arrows.

Samples were collected during two regional voyages in the south-eastern Indian Ocean aboard the RV Southern Surveyor (SS) in August (SS2012_V04) and September (2012 SS2012_T06; Fig 2). No additional specific permissions were required for any of the voyages. Associated biogeochemical meta and underway data can be downloaded from http://www.imos.org.au/. Associated biogeochemical meta and underway data can be downloaded from http://www.imos.org.au/. For DNA analysis, 2 L of seawater was filtered through Sterivex capsules (0.2 μm pore size) with a peristaltic pump. A modified organic (phenol:chloroform:isoamyl based) DNA extraction protocol was used alongside extraction columns from the PowerWater DNA isolation kit (Mo Bio Laboratories, USA). DNA extraction protocol has been described in Raes et al. [21]. Functional genes encoding for nitrous oxide reductase (*nosZ*) were quantified in technical triplicates by real-time PCR (qPCR) using a 7500 real-time PCR system (Applied Biosystems, Foster City, USA). *nosZ* genes were amplified using *nosZ*-F and *nosZ* 1622R primers [22]. The 15-μl reactions contained 0.15μL 100x BSA, 0.1 μL forward and reverse primers,7.5 μl 2 x SensiFAST™ master mix and 2 μL template DNA (final concentration between 10-25ng template DNA). Cycling conditions were 1 cycle at 96°C for 3 min, followed by 40 cycles at 96°C for 45s, followed by 30s at the annealing temperature of 55°C, followed by 30s at 72°C and 34s at the fluorescent acquisition temperature of 82°C. Dissociation curves were run at 95°C for 15s, followed by 1 min at 55°C and 15s at 95°C. Standards for *nosZ* gene quantification using qPCR were prepared by amplifying a constructed plasmid containing the respective gene fragment from an environmental clone. Standards for qPCR were made up using a serial dilution (10^−1^>>10^−6^ ng.μL^-1^) of known copies of PCR fragments.

Polymerase chain reactions (PCR) for h*zsA* genes were set up using *hzsA*1597F and *hzsA*1857R [23]. Cycling conditions were 1 cycle at 96°C for 3 min, followed by 40 cycles at 96°C for 45s, followed by 30s at the annealing temperature of 55°C, followed by 30s at 72°C.

PCR products were purified from the reaction mix using magnetic beads (Agincourt, Beverly, MA, USA).Clone libraries were setup using the Invitrogen TOPO TA Cloning® Kit according to the manufacturer’s instructions. *nosZ* and *hzsA* gene fragments were sequenced using an ABI 3130XL genetic analyzer (Applied Biosystems) and aligned using Geneious® and Arb software package (http://www.arb-home.de/). Total microbial abundance was measured using a Beckman Coulter Gallios flow cytometer counter and has been described in detail in Raes et al. [21] (S1 Table).

### Argo data

Oxygen data from 229 Argo float profiles were analysed from 2006 up to September 2014. These data were collected and made freely available by the International Argo Program and the national programs that contribute to it (http://www.argo.ucsd.edu, http://argo.jcommops.org). Argo floats numbers used were 4900483; 4900484; 4900485; 4900487; 4900487; 4900441; 5901310; 5901311; 5901313; 5901314; 5901369; 5901646; 5901697; 5902100; 5902105; 5903593. The Argo Program is part of the Global Ocean Observing System. Argo float data were sourced from the Integrated Marine Observing System (IMOS; http://imos.org.au/). Additional information on the oxygen data within the Argo data system can be found in the documentation of Processing Argo Oxygen data at the DAC level (http://www.argodatamgt.org/Documentation). For completion on the QC of the Argo data we note that Takeshita et al. [24] have reported a mean oxygen sensor error, relative to the World Ocean Atlas climatology data, of about 10 μmol.kg^-1^ in surface waters with sensors generally reading too low.

## Results and Discussion

Here we present results describing the presence of the nitrous oxide reductase enzyme (*nosZ*) in cruise samples collected from well-oxygenated (180–250 μmol kg^−1^) photic zone waters in the south-eastern Indian Ocean. We detected the presence *nosZ* genes with an average concentrations of 1.9x10^5^±1.31x10^5^ *nosZ* copies mL^-1^ (±SD, n = 18). Our data compliment the *nosZ* gene copy concentrations ranging between ~1 x 10^3^ to 1 x 10^5^ copies mL^-1^ at the oxygenated surface waters and the deeper hypoxic waters in the Arabian Sea reported by Wyman et al. [25]. These combined results show a wide biogeographical distribution of *nosZ* genes in both south-eastern and western parts of the Indian Ocean. Bacterial clone sequencing of the *nosZ* genes in the south-eastern Indian Ocean showed an overall dominance of *Rhodobacteraceae*, with the majority of the sequences belonging to uncultured nitrous oxide reductase bacteria clones 3–57 *nosZ*, 31-*nosZ*, 29-*nosZ*-LZB and 39-*nosZ*-LZB.

**Fig 3:**
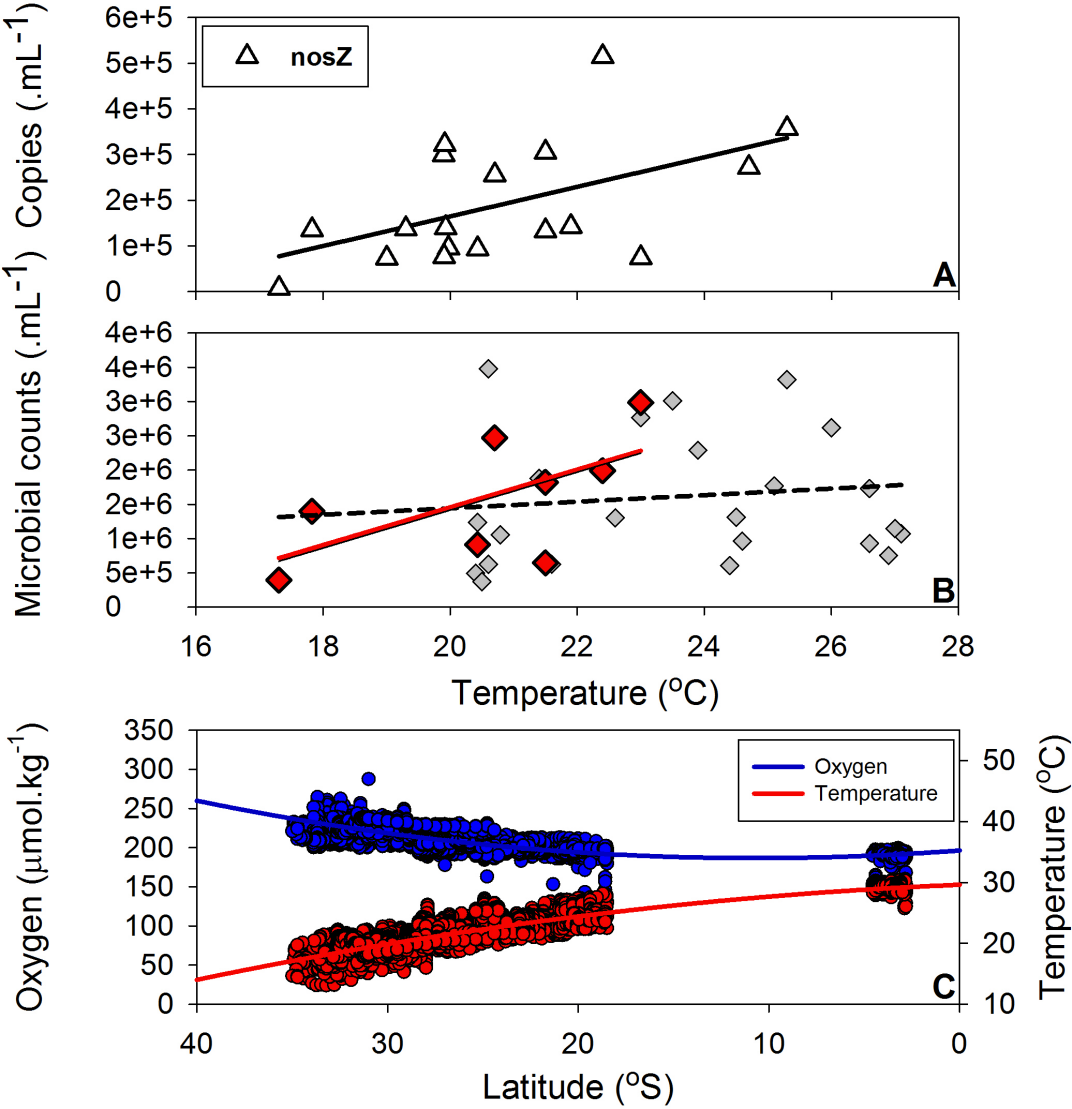
(A) Nitrous oxide reductase (*nosZ*) gene copies (copies mL^-1^) plotted against in situ temperature data (°C); *nosZ*: r^2^ = 0.27, slope = 32381 copies.°C^-1^, coefficient standard error = 13259, p = 0.027. (B) Microbial counts (cells mL^-1^) versus in situ temperature data; red diamonds denote stations where *nosZ* genes were measured. These microbial counts suggest a positive correlation with temperature (r^2^ = 0.39, p = 0.098). Grey diamonds present regional microbial abundance but do not suggest a temperature correlation (r^2^ = 0.016, p = 0.5); see Fig 2 for spatial coverage. Fig 3 C presents a latitudinal relationship of oxygen (r^2^ = 0.6, p<0.0001) and temperature (r^2^ = 0.8, p<0.0001). Data are capped from the surface to 50 m depth and sourced from all available Argo floats through the Australian Argo DAC as part of the IMOS portal and cruise voyages conducted on the Southern Surveyor in 2010, 2011, 2012 and 2013.

Our *nosZ* gene copy data correlated positively with increasing temperatures in the south-eastern Indian Ocean (Fig 3A). The total microbial abundance however showed no significant relationship with temperature and regionally averaged ~1.6 x 10^6^ cells mL^-1^ (n = 31; Fig 3B, Fig 2 and S1 Table). Across the data set, a conservative estimate suggests that the organisms catalysing the reduction of N_2_O to the inert N_2_ gas could represent over 1% of the heterotrophic cell abundance (assuming up to 5 *nosZ* copies per cell), which is a significant proportion of the functional microbial community. Yet, these percentage drastically increase when we assume lower *nosZ* copy numbers per cell [26]. The positive environmental gradient of *nosZ* gene copy numbers and temperature, along with a decline in oxygen (Fig 3C and S1 Fig), suggests a preferential nitrous oxide reductase niche at higher temperatures and a potential and important sink for the harmful N_2_O gas in the warmer Tropical waters. Butler et al. [27] measured supersaturation of N_2_O around 20% with a maximum of 37%, near 8°S in the eastern Indian Ocean. The authors were able to link this supersaturation of N_2_O to upwelling events near the boundary of the equatorial counter current and the south equatorial current [28]. Dissolved N_2_O concentrations at depth in the eastern Indian Ocean have been reported by the former authors to be higher in the northern latitudes, where they have been shown to form a core of high N_2_O (~150-600m) north of the equator [27].

Raes et al. [21] showed that along the same temperature gradient, shown here, the total dissolved inorganic nitrogen (DIN) pool increased significantly from the subtropics (35°S) to the tropics (12°S), and that the highest DIN concentrations occurred at the highest NH_4_^+^:NO_3_^−^ ratios. Along this latitudinal gradient the authors also noted that the microbial community in the subtropics (cooler waters) were associated with deep nutrient fluxes (preferential NO_3_^−^ and PO_4_^3−^ concentrations) and that the microbial community in the tropics (warmer waters) were linked with an increase in NH_4_^+^ and NO_2_^-^ concentrations. The Tropical warmer waters are shown to be subjected to rapid recycling of organic matter where primary productivity is controlled via ammonification and nitrification within the euphotic zone [21]. The positive slope between *nosZ* copy numbers and increasing temperature indicates a feedback between the production (nitrification) and reduction of N_2_O. Large blooms of *Trichodesmium* also occur in these warm Tropical waters. *Trichodesmium* spp. has been suggested as a potential host for denitrifying bacteria in oxygenated waters of the Arabian Sea [25]. The low oxygen habitats within *Trichodesmium* colonies and other marine aggregates are interesting niches for cryptic N-cycling process, and are under explored habitats for a range of N cycling genes.

Pearce and Feng [29] confirmed a warming trend of ~0.02°C year^–1^ in the south-eastern Indian Ocean since 1951 from *in situ* temperature measurements at a coastal monitoring station on the Western Australian continental shelf. Marine heat waves such as the one recorded in 2011 in the south-eastern Indian Ocean are linked with El Niño/Southern Oscillations and are predicted to increase in frequency as a result of global warming [30,31], yet their ecological impacts towards primary productivity are not well understood [32]. The increasing *nosZ* gene abundance with temperature raises the question of whether increased nitrification rates and the consequent enhanced production of N_2_O through extreme climatic warming events and warming sea surface temperatures could positively be balanced by the reduction of N_2_O.

**Fig 4:**
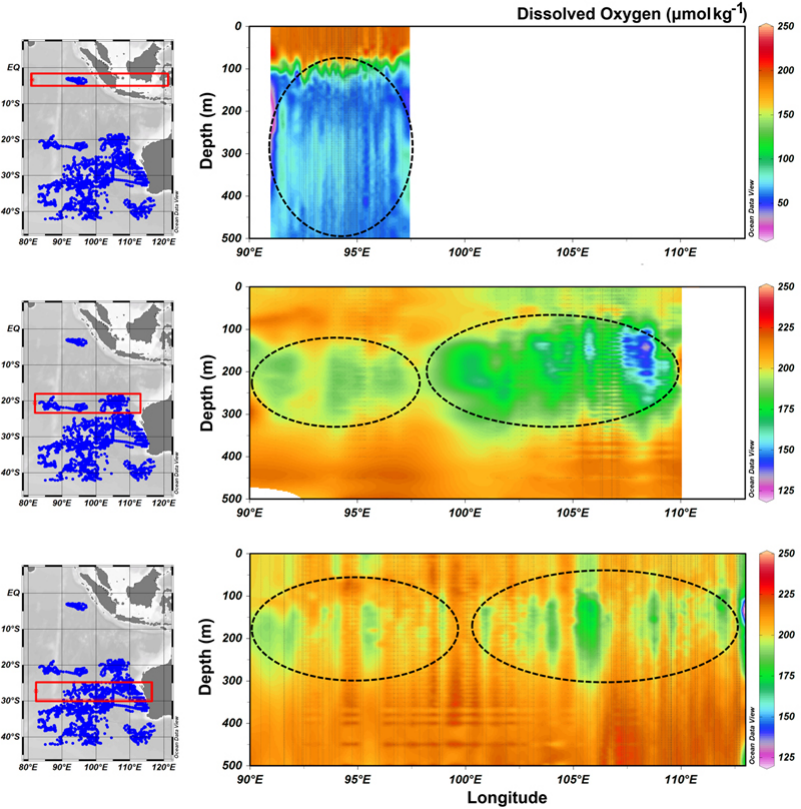
Spatial extent of shallow (100-200m) low dissolved oxygen layers stretching from the tropics to the subtropics. Data (blue dots) sourced from all available ARGO floats with oxygen in the region and cruise voyages conducted on the Southern Surveyor in 2010, 2011, 2012 and 2013. The red rectangle indicates the latitudinal area where a longitudinal slice through the water column has been analysed. Low dissolved oxygen layers (μmol kg^-1^) are highlighted by black circles.

Oceanic oxygen concentrations impact a suite of biogeochemical cycling parameters that will influence N-cycling processes and carbon sequestration in the tropical oceans [33]. Thompson et al (2011) proposed that shallow (100-200m) lower dissolved oxygen layers (~180 μmol kg^−1^) in the south-eastern Indian Ocean ~32°S are physically continuous with similar layers as far north as 6°S in the North Indian Ocean (~50 μmol kg^−1^). The analysis of 229 vertical Argo floats profiles and *in situ* oxygen data from 4 voyages in the south-eastern Indian Ocean confirmed this conceptual model (Figs 2–4). The tight linkage between N and O is further described by the conservative water mass tracer “NO” [14] where respiration of organic matter and O_2_ consumption are combined into a single parameter. Waite et al. [13] and Raes et al. [19] proposed that these lower oxygenated waters are a hotspot for a diverse range of N cycling processes that play a vital role in providing necessary inorganic N compounds through ammonification and nitrification thereby sustaining primary productivity in these oligotrophic waters. In this data set we show that *nosZ* gene copy numbers correlated with these lower “NO” and lower oxygenated waters.

To date, little information is available on the potential feedback between the reduction of N_2_O and denitrification (bio available N losses) outside oxygen minimum zones in this and many other regions of the global ocean. Nitrous oxide reductase does not catalyse denitrification, as denitrification is defined as the conversion of fixed/reactive nitrogen to N_2_O. We can therefore not assume a priori that organisms that carry *nosZ* genes also have other N reduction genes such as *nir* and *nor* genes. Yet, the dominance of *Rhodobacteraceae* in our samples suggests that the intermediate steps in the denitrification pathway (from NO_3_^-^ to NO_2_^-^ and NO) could also be present [34]. Li et al. [35] also shown a close coupling between NO_3_^-^ deficits and active denitrification in the Indian Ocean. Many authors have highlighted a close coupling between N_2_ fixation and denitrification [36–38] while others have shown a significant correlation in the abundance of *nosZ* genes and denitrification rates [39–41]. The south-eastern Indian Ocean has relatively higher N_2_ fixation rates compared to the Atlantic and Pacific [21], which suggest a potential for denitrification rates. Furthermore, aerobic denitrification has theoretically been suggested [42] and empirically been shown [43] in the marine environment. A number of authors [44–46] have also highlighted active N losses associated with suboxic and anaerobic microhabitats, such as biofilms on marine aggregates in generally oxygenated waters. We therefore postulate that the south-eastern Indian Ocean is also subjected to denitrification (bio-available N-losses) in the photic zone. Most studies report a relationship between N loss gene copy numbers and N loss rates. In the OMZ waters of the Arabian Sea and the Peruvian upwelling, Jayakumar et al. [47] reported a range of denitrification gene copies from 2 to 6 x10^5^ mL^-1^ which related to N_2_ loss rates up to 26 nmol.L^-1^ of N_2_ day^-1^. Although we lack rates for the above processes, the abundance of *nosZ* gene copies from our results are an interesting finding and the potential implications for bio available N-losses are worth further investigation.

It is crucial that we keep the biogeography principle in mind of: “Everything is everywhere but nature selects” [48]. Yet, we note that in all our samples we detected hydrazine synthase (*hzsA*) genes, which are the functional encoders for the anammox process. Clone sequencing from PCR products revealed that clones belonged to the genera Candidatus *Scalindua*, *Jettenia* and the species *Brocadia fulgida*. Uncultured anaerobic ammonium-oxidizing bacterial clones were also detected in our samples and closest sequence belonged to clones BS21, clone jwl2F/2R and clone I230-3. The finding of the anammox process in detectable quantities in these oxygenated waters is surprising and a novel finding.

## Conclusion

The reduction of the potent greenhouse gas N_2_O to the inert N_2_ gas is of a global concern. Here we showed that the Tropical waters of the south-eastern Indian Ocean can act as a potential sink for N_2_O. Our data suggest a close coupling between nitrification (the production of N_2_O) and denitrifiers (the reduction of N_2_O). We propose that the lower oxygenated waters of the south-eastern Indian Ocean can both act as a sink and a source of N_2_O emissions. As a new testable hypothesis we suggest that we are underestimating N-losses in this and many other marine ecosystems. Future work which would allow us to fully understand the ecological role of the *nosZ* gene and even anammox bacteria in the lower oxygenated surface water of the south eastern Indian Ocean could include stable isotope probing [49], reverse transcriptive activities of *nosZ* genes [50] and quantification of nitrification and denitrification rates [51]. The inclusion of stations from the OMZ in the Arabian Sea would allow a greater understanding of the sink and source terms of N_2_O [52] and *nosZ* gene activity under aerobic and anaerobic environments within the same Ocean basin. In summary our data highlights the need to further investigate key process in the marine nitrogen cycle in this understudied yet strategic Ocean basin as sink and source terms are predicted to alter under global change scenarios such as ocean warming and decreasing oxygen concentrations.

## Supporting Information

S1 Fig Dissolved oxygen vs nitrite (NO_2_^_^) concentrations in the south-eastern Indian Ocean. See Fig 1 for CTD stations. Note: Elevated NO_2_^_^ concentrations up to 0.3 μmol.L^-1^ in relative oxygenated surface waters.
(TIF)

S1 Table Metadata for microbial community data. (DOCX)
