# Supplementary figures and images for: Reduction of the Powerful Greenhouse Gas N2O in the South-Eastern Indian Ocean
Source: PLoS One. 2016 Jan 22;11(1):e0145996. doi: 10.1371/journal.pone.0145996 (PMC4723335; doi:10.1371/journal.pone.0145996)

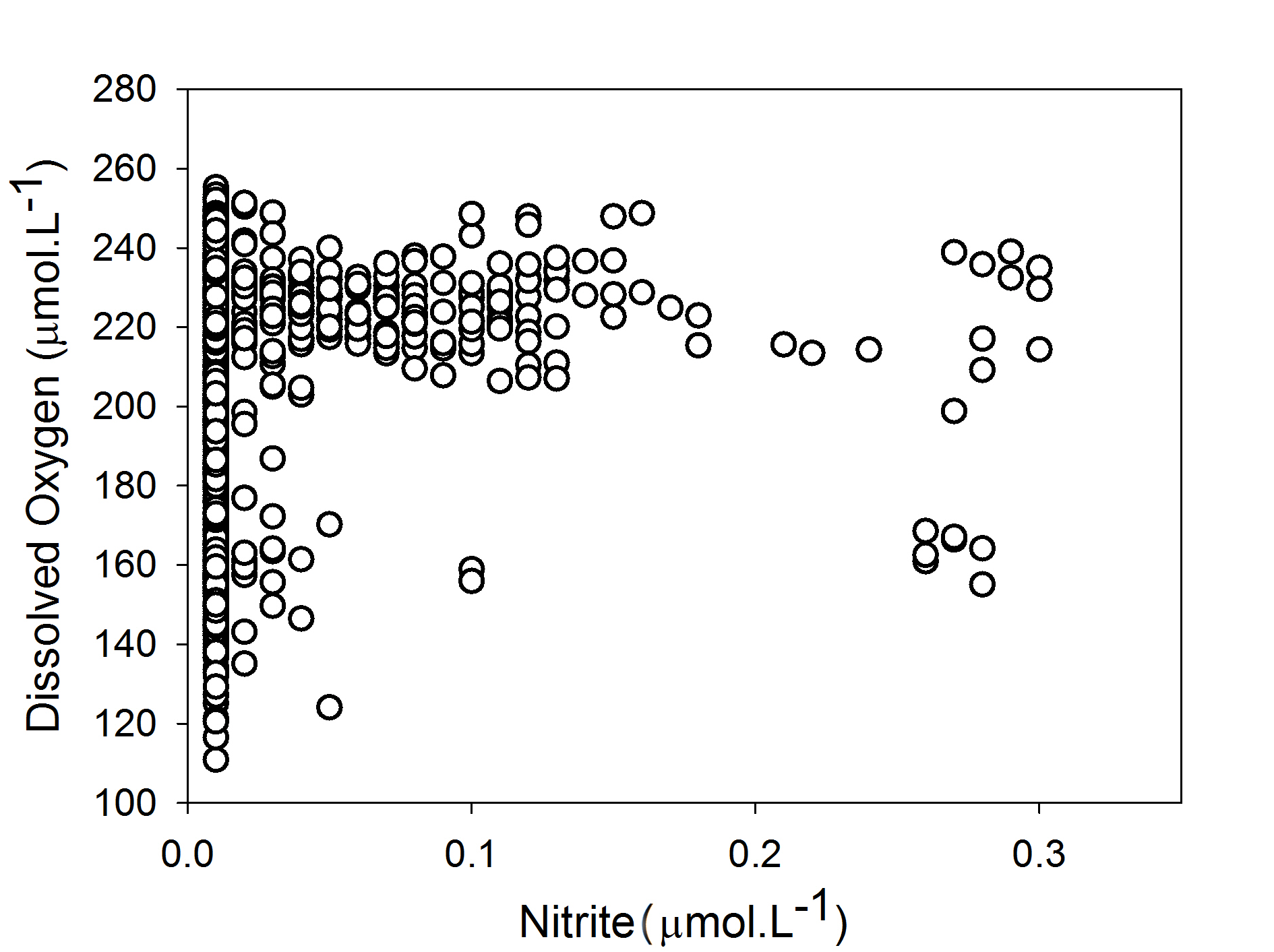

Supplement: S1 Fig — See Fig 1 for CTD stations. Note: Elevated NO2_ concentrations up to 0.3 μmol.L-1 in relative oxygenated surface waters. (TIF) [file pone.0145996.s001.tif]
