# Supplementary material for: Reduction of the Powerful Greenhouse Gas N2O in the South-Eastern Indian Ocean
Source: PLoS One. 2016 Jan 22;11(1):e0145996. doi: 10.1371/journal.pone.0145996 (PMC4723335; doi:10.1371/journal.pone.0145996)
Supplement: S1 Table — (DOCX) [file pone.0145996.s002.docx]

**Supporting information**

**Reduction of the powerful greenhouse gas N_2_O in the south-eastern Indian Ocean**

Eric J. Raes^1,3*^, Levente Bodrossy^2^, Jodie Van de Kamp^2^, Bronwyn Holmes^2^, Nick Hardman-Mountford^3,1^, Peter A. Thompson^2^, Allison S. McInnes^4^, Anya M. Waite^5^

**S1 Table 1. Metadata for microbial community data.**

| **Voyage** | **Station** | **Depth** | **Latitude (˚S)** | **Longitude (˚E)** | **Salinity PSU (at sampling depth)** | **Temperature ˚C (at sampling depth)** | **Microbes (cells mL^-1^)** | **nosZ copies mL^-1^** |
| --- | --- | --- | --- | --- | --- | --- | --- | --- |
| **SS2012 V04** | CDT6 | O_2_ min | -32.5977 | 114.5417 | 35.34 | 19.97 |  | 95614.58 |
| **SS2012 V04** | CDT6 | SFC | -32.5977 | 114.5417 | 35.22 | 19.93 |  | 139991.89 |
| **SS2012 V04** | CDT7 | SFC | -32.5847 | 114.4703 | 35.22 | 19.89 |  | 299967.31 |
| **SS2012 V04** | CDT10 | SFC | -32.5273 | 114.1502 | 35.24 | 19.91 |  | 321902.42 |
| **SS2012 V04** | FT1 | SFC | -31.9068 | 115.4497 | 35.23 | 19.3 |  | 137485.02 |
| **SS2012 V04** | FT3 | SFC | -31.1922 | 113.845 | 35.43 | 18.9 |  | 137485.02 |
| **SS2012 V04** | CDT30 | O_2_ min | -31.0013 | 105.0018 | 35.76 | 17.31 | 395368.42 | 7236.71 |
| **SS2012 V04** | FT6 | SFC | -30.178 | 111.5898 | 35.11 | 20.7 | 2469636.36 | 255722.13 |
| **SS2012 V04** | CDT27 | O_2_ min | -29.4999 | 105.0002 | 35.77 | 17.83 |  | 135672.72 |
| **SS2012 V04** | CDT27 | O_2_ min | -29.4999 | 105.0002 | 35.77 | 17.83 | 1400751.30 |  |
| **SS2012 V04** | CDT26 | SFC | -28.9974 | 104.9986 | 35.29 | 20.43 | 1237497.33 | 93989.75 |
| **SS2012 V04** | FT17 | SFC | -27.2568 | 105.2198 | 35.07 | 21.5 | 1820541.79 | 133297.97 |
| **SS2012 V04** | FT23 | SFC | -26.3085 | 103.1885 | 34.98 | 21.5 | 646191.30 | 305341.73 |
| **SS2012 V04** | CDT19 | O_2_ min | -25.9998 | 104.9992 | 35.16 | 21.38 |  | 76991.61 |
| **SS2012 V04** | CDT17 | SFC | -24.9998 | 105.0006 | 35.15 | 21.38 |  | 514631.42 |
| **SS2012 T06** | D1 | SFC | -31.6775 | 115.1651 | 35.58 | 19 |  | 73741.96 |
| **SS2012 T06** | D6 | SFC | -29.3464 | 113.4344 | 35.08 | 20.6 | 625337.60 |  |
| **SS2012 T06** | D8 | SFC | -28.5857 | 112.9717 | 35 | 20.79 | 1058060.15 |  |
| **SS2012 T06** | D9 | SFC | -28.1708 | 112.8139 | 35.2 | 20.4 | 494912.52 |  |
| **SS2012 T06** | D10 | SFC | -27.5401 | 112.5761 | 35.17 | 20.6 | 3480031.65 |  |
| **SS2012 T06** | D13 | SFC | -26.1294 | 112.0482 | 35.03 | 20.5 | 370867.85 |  |
| **SS2012 T06** | D14 | SFC | -25.6509 | 112.0001 | 35.07 | 21.4 | 1881399.07 |  |
| **SS2012 T06** | D15 | SFC | -25.1493 | 112.0002 | 35.06 | 21.6 | 625567.52 |  |
| **SS2012 T06** | D16 | SFC | -24.6411 | 112 | 34.89 | 22.6 | 1303908.85 |  |
| **SS2012 T06** | D17 | SFC | -23.9808 | 112.0118 | 34.93 | 22.4 | 1997355.06 | 514631.42 |
| **SS2012 T06** | D20 | SFC | -22.6941 | 112.7855 | 34.8 | 23 | 2766649.95 | 74241.91 |
| **SS2012 T06** | D23 | SFC | -21.4645 | 113.519 | 34.8 | 23.9 | 2291175.93 |  |
| **SS2012 T06** | D24 | SFC | -20.9715 | 113.9577 | 34.74 | 23.5 | 3009051.61 |  |
| **SS2012 T06** | D27 | SFC | -19.7644 | 115.1464 | 34.64 | 24.4 | 606780.05 | 74241.91 |
| **SS2012 T06** | D28 | SFC | -19.3276 | 115.6252 | 34.5 | 24.5 | 1312950.86 |  |
| **SS2012 T06** | D29 | SFC | -18.9044 | 116.2185 | 34.79 | 24.6 | 962731.86 |  |
| **SS2012 T06** | D32 | SFC | -18.1158 | 117.3202 | 34.37 | 25.3 |  | 271970.36 |
| **SS2012 T06** | D34 | SFC | -17.5769 | 118.0668 | 34.44 | 25.1 | 1768306.11 | 356961.10 |
| **SS2012 T06** | D35 | SFC | -17.0068 | 118.8859 | 34.45 | 25.3 | 3318392.69 |  |
| **SS2012 T06** | D37 | SFC | -16.323 | 119.9417 | 34.33 | 26 | 2619529.31 |  |
| **SS2012 T06** | D40 | SFC | -15.3743 | 121.3999 | 34.18 | 26.6 | 929229.79 |  |
| **SS2012 T06** | D41 | SFC | -15.0758 | 121.8571 | 34.21 | 26.6 | 1730027.78 |  |
| **SS2012 T06** | D42 | SFC | -14.792 | 122.2913 | 34.27 | 26.9 | 756193.95 |  |
| **SS2012 T06** | D43 | SFC | -14.3889 | 122.9069 | 34.16 | 27.1 | 1070499.33 |  |
| **SS2012 T06** | D45 | SFC | -14.067 | 123.3981 | 34.36 | 27 | 1147827.07 |  |
| **SS2012 T06** | D49 | SFC | -12.7122 | 125.6473 | 34.04 | 27.3 | 1588786.54 |  |

SFC =Surface; O_2_ min between 90 and 175m depth.

Table 1. Metadata for microbial community data.

| **Voyage** | **Station** | **Depth** | **Latitude (˚S)** | **Longitude (˚E)** | **Salinity PSU (at sampling depth)** | **Temperature ˚C (at sampling depth)** | **Microbes (cells mL^-1^)** | **nosZ copies mL^-1^** |
| --- | --- | --- | --- | --- | --- | --- | --- | --- |
| **SS2012 V04** | CDT6 | O_2_ min | -32.5977 | 114.5417 | 35.34 | 19.97 |  | 95614.58 |
| **SS2012 V04** | CDT6 | SFC | -32.5977 | 114.5417 | 35.22 | 19.93 |  | 139991.89 |
| **SS2012 V04** | CDT7 | SFC | -32.5847 | 114.4703 | 35.22 | 19.89 |  | 299967.31 |
| **SS2012 V04** | CDT10 | SFC | -32.5273 | 114.1502 | 35.24 | 19.91 |  | 321902.42 |
| **SS2012 V04** | FT1 | SFC | -31.9068 | 115.4497 | 35.23 | 19.3 |  | 137485.02 |
| **SS2012 V04** | FT3 | SFC | -31.1922 | 113.845 | 35.43 | 18.9 |  | 137485.02 |
| **SS2012 V04** | CDT30 | O_2_ min | -31.0013 | 105.0018 | 35.76 | 17.31 | 395368.42 | 7236.71 |
| **SS2012 V04** | FT6 | SFC | -30.178 | 111.5898 | 35.11 | 20.7 | 2469636.36 | 255722.13 |
| **SS2012 V04** | CDT27 | O_2_ min | -29.4999 | 105.0002 | 35.77 | 17.83 |  | 135672.72 |
| **SS2012 V04** | CDT27 | O_2_ min | -29.4999 | 105.0002 | 35.77 | 17.83 | 1400751.30 |  |
| **SS2012 V04** | CDT26 | SFC | -28.9974 | 104.9986 | 35.29 | 20.43 | 1237497.33 | 93989.75 |
| **SS2012 V04** | FT17 | SFC | -27.2568 | 105.2198 | 35.07 | 21.5 | 1820541.79 | 133297.97 |
| **SS2012 V04** | FT23 | SFC | -26.3085 | 103.1885 | 34.98 | 21.5 | 646191.30 | 305341.73 |
| **SS2012 V04** | CDT19 | O_2_ min | -25.9998 | 104.9992 | 35.16 | 21.38 |  | 76991.61 |
| **SS2012 V04** | CDT17 | SFC | -24.9998 | 105.0006 | 35.15 | 21.38 |  | 514631.42 |
| **SS2012 T06** | D1 | SFC | -31.6775 | 115.1651 | 35.58 | 19 |  | 73741.96 |
| **SS2012 T06** | D6 | SFC | -29.3464 | 113.4344 | 35.08 | 20.6 | 625337.60 |  |
| **SS2012 T06** | D8 | SFC | -28.5857 | 112.9717 | 35 | 20.79 | 1058060.15 |  |
| **SS2012 T06** | D9 | SFC | -28.1708 | 112.8139 | 35.2 | 20.4 | 494912.52 |  |
| **SS2012 T06** | D10 | SFC | -27.5401 | 112.5761 | 35.17 | 20.6 | 3480031.65 |  |
| **SS2012 T06** | D13 | SFC | -26.1294 | 112.0482 | 35.03 | 20.5 | 370867.85 |  |
| **SS2012 T06** | D14 | SFC | -25.6509 | 112.0001 | 35.07 | 21.4 | 1881399.07 |  |
| **SS2012 T06** | D15 | SFC | -25.1493 | 112.0002 | 35.06 | 21.6 | 625567.52 |  |
| **SS2012 T06** | D16 | SFC | -24.6411 | 112 | 34.89 | 22.6 | 1303908.85 |  |
| **SS2012 T06** | D17 | SFC | -23.9808 | 112.0118 | 34.93 | 22.4 | 1997355.06 | 514631.42 |
| **SS2012 T06** | D20 | SFC | -22.6941 | 112.7855 | 34.8 | 23 | 2766649.95 | 74241.91 |
| **SS2012 T06** | D23 | SFC | -21.4645 | 113.519 | 34.8 | 23.9 | 2291175.93 |  |
| **SS2012 T06** | D24 | SFC | -20.9715 | 113.9577 | 34.74 | 23.5 | 3009051.61 |  |
| **SS2012 T06** | D27 | SFC | -19.7644 | 115.1464 | 34.64 | 24.4 | 606780.05 | 74241.91 |
| **SS2012 T06** | D28 | SFC | -19.3276 | 115.6252 | 34.5 | 24.5 | 1312950.86 |  |
| **SS2012 T06** | D29 | SFC | -18.9044 | 116.2185 | 34.79 | 24.6 | 962731.86 |  |
| **SS2012 T06** | D32 | SFC | -18.1158 | 117.3202 | 34.37 | 25.3 |  | 271970.36 |
| **SS2012 T06** | D34 | SFC | -17.5769 | 118.0668 | 34.44 | 25.1 | 1768306.11 | 356961.10 |
| **SS2012 T06** | D35 | SFC | -17.0068 | 118.8859 | 34.45 | 25.3 | 3318392.69 |  |
| **SS2012 T06** | D37 | SFC | -16.323 | 119.9417 | 34.33 | 26 | 2619529.31 |  |
| **SS2012 T06** | D40 | SFC | -15.3743 | 121.3999 | 34.18 | 26.6 | 929229.79 |  |
| **SS2012 T06** | D41 | SFC | -15.0758 | 121.8571 | 34.21 | 26.6 | 1730027.78 |  |
| **SS2012 T06** | D42 | SFC | -14.792 | 122.2913 | 34.27 | 26.9 | 756193.95 |  |
| **SS2012 T06** | D43 | SFC | -14.3889 | 122.9069 | 34.16 | 27.1 | 1070499.33 |  |
| **SS2012 T06** | D45 | SFC | -14.067 | 123.3981 | 34.36 | 27 | 1147827.07 |  |
| **SS2012 T06** | D49 | SFC | -12.7122 | 125.6473 | 34.04 | 27.3 | 1588786.54 |  |

SFC =Surface; O_2_ min between 90 and 175m depth.
